# Vesicovaginal fistulae

**DOI:** 10.4103/0970-1591.65400

**Published:** 2010

**Authors:** Mary Garthwaite, Neil Harris

**Affiliations:** Pyrah Department of Urology, Leeds Teaching Hospitals NHS Trust, Leeds, LS9 7TF, UK

**Keywords:** Vesicovaginal fistula, pressure necrosis, management

## Abstract

Vesicovaginal fistula (VVF) formation represents a condition with devastating consequences for the patient and continues to pose a significant challenge to the surgeon. Quick and accurate diagnosis, followed by timely repair is essential to the successful management of these cases. A thorough understanding of the pathophysiology and anatomy of the fistula, potential factors that may compromise healing and experience in the fundamental principles of fistula repair are the vital tools of the fistula surgeon. This review was undertaken to provide an overview of the key areas in VVF investigation and management.

## INTRODUCTION

Regardless of the etiopathology, the development of a vesicovaginal fistula (VVF) has profound and devastating consequences for the patient’s physical and psychological health. First reports of successful repairs emerged in the literature around the mid-19^th^ century when James Marion Sims described his technique of a transvaginal approach with the use of silver sutures and bladder drainage postoperatively.[1] Medical advances allowing transabdominal surgery heralded the development of modern-day fistula repair techniques. However, despite these advances VVF repair continues to present a major technical challenge for modern surgery.

## ETIOLOGY

Worldwide, prolonged, obstructed labor is the leading cause of VVF. This, however, is an uncommon occurrence in the developed world, largely due to the availability of advanced obstetric practice.

In West Africa VVF is estimated to occur in up to three in 100,000 deliveries. Factors influencing this rate include young maternal age (physical immaturity of the mother’s body leads to cephalopelvic disproportion), a paucity of modern obstetric facilities and skills and ‘traditional’ practices such as female circumcision. In the developed world approximately 90% of VVF are secondary to accidental injury to the bladder during surgery. High-risk procedures include hysterectomy (75% of cases) and urological or lower gastrointestinal pelvic surgery (^~^2%).[2,3] In general, risk factors for VVF formation include previous uterine surgery, pelvic irradiation, endometriosis and anatomical distortion (such as a large fibroid uterus). A compounding factor is one that will compromise healing, such as anemia, malnutrition and steroid use. Other less common causes include pelvic malignancy, obstetric infections, erosion secondary to a foreign body and vaginal trauma.

## PATHOGENESIS

In prolonged, obstructed labor pressure necrosis of the anterior vaginal wall and underlying bladder neck or urethra occurs as the tissues are compressed between the fetal head and the posterior surface of the symphysis pubis. When the mother survives, the necrotic tissue sloughs away and is expelled after approximately 10 days, at which point incontinence ensues.

VVF following hysterectomy is most likely to arise from an unrecognized bladder injury at the time of surgery, which results in the formation of a urinoma. Urine will then follow the path of least resistance and drain through the vaginal cuff suture line. A mucosa-lined tract will then establish between the bladder and the vagina. A second possible mechanism is pressure necrosis from incorrectly placed sutures between the vaginal cuff and posterior bladder. Hematoma and infection are often complicating factors.

## PRESENTATION AND ASSESSMENT

The majority of VVF present with leakage of urine per vagina at approximately 5-10 days postoperatively. Occasionally, urine leakage becomes apparent in the immediate postoperative period. The possibility of unrecognized intra-operative injury to the urinary tract, and hence an impending fistula, should be considered in any patient with a prolonged ileus, excessive pain or hematuria following pelvic surgery.

VVF can also develop at a distant interval after pelvic irradiation or secondary to local malignancy, with some reports of up to 20 years between radiotherapy and fistula formation.

The differential diagnosis of a VVF encompasses ureterovaginal fistula, uterovaginal fistula, peritoneal fluid, vaginal discharge secondary to infection and pelvic abscess or collection. Biochemical analysis of the fluid can help differentiate urine from serous fluid. Direct visualization and assessment of the defect on vaginal and cystoscopic examination is helpful in planning surgical repair. The use of methylene blue solution intravesically can assist in determining the site of the fistula. Ten to fifteen per cent of all VVF have been found to have coexistent ureteric injury[3] and therefore assessment of the upper urinary tract is mandatory with either intravenous urogram (IVU) or retrograde pyelography at the time of cystoscopic examination.

## CONSERVATIVE MANAGEMENT

Conservative management of a small VVF relies on spontaneous closure of the defect during a period of catheter drainage of the bladder. Its success has been reported in small numbers of cases following hysterectomy, with catheterization times ranging from 19-54 days.[4]

Several minimally invasive approaches for the treatment of small fistulae have been reported. Injection of a fibrin-based glue into the fistula tract was first reported by Pettersson in 1979.[5] Fibrin glue promotes healing via effects on fibroblasts and collagen synthesis and has the advantage of being biodegradable. Over the years there have been several reports of success with this technique, however the number of patients treated is small and the size of the fistula tract is often tiny.[6–8] Electrocoagulation of the fistula tract, either transvaginally or transurethrally has been employed successfully in small numbers of patients with fistulas less than 3 mm in diameter.[9] The technique involves the use of a tiny electrode and minimal coagulation current so that the risk of increasing the size of the fistula is kept to a minimum.

There is currently no consensus on the time that should be allotted for spontaneous healing to occur and when to abandon conservative treatment and consider surgical repair. A period of catheterization prior to surgical correction is common and does offer a degree of symptomatic relief to the patient. Traditionally, VVF repair has been deferred for three to six months to allow inflammation and edema to settle. This is certainly applicable to the management of large, complex fistulae. However, early repair of simple, iatrogenic fistulae has been found to be highly successful and minimizes the physical and psychological disruption to the patient’s life.

## SURGICAL REPAIR

Regardless of the technique used and the timing of surgery, the principles that underpin VVF repair remain the same. The repair should be tension-free, watertight and uninfected. The tissues at the site of the repair should be healthy and a well-vascularized interposition flap should be used if required. The first attempt at VVF repair has the highest chance of success, making it imperative that surgery is well planned and performed by a surgeon experienced in fistula repair.

VVF repair can be approached transvaginally, transabdominally, or in a combined approach if necessary. The transvaginal approach offers a lower complication rate and a shorter postoperative recovery. The transabdominal route is preferred when the fistula site cannot be visualized or easily accessed per vagina, or when the VVF is complex.

## TRANSVAGINAL APPROACH

The transvaginal approach offers the advantages of avoidance of laparotomy and opening of the bladder, minimal blood loss, rapid postoperative recovery and shorter hospital stay.[10] Success rates have been shown to be equivalent to those for transabdominal repair.[11]

The vaginal flap technique is the most commonly used method of repair.[12] This utilizes a ‘U’-shaped flap with the fistula at the base of the ‘U’. The fistula is then circumscribed and the vaginal wall mobilized away from it. The vaginal wall beyond the fistula is excised to allow advancement of the vaginal flap so that the line of closure will be sited away from the underlying fistula defect. There is continued debate as to whether the fistula tract should be excised or not.[10,13] The concern is that excision will increase the size of the fistulous defect and potentially decrease the strength of the repair. The fistula is closed transversely taking care to invert the bladder mucosa and to avoid the ureters. A second layer of sutures strengthens the bladder wall. A Martius flap, formed from a labial fat pad, can be used to provide a layer of healthy, vascularized tissue between the fistula repair and the vaginal wall. This reinforces the closure in cases where the viability of the tissues is doubtful and increases the success of the repair. However, the use of an interposition flap is not necessary if the fistula is small and uncomplicated.[14]

## TRANSABDOMINAL APPROACH

A transabdominal approach to fistula repair is indicated if the vaginal exposure of the fistula defect is inadequate (high vaginal defect, retracted defect or narrow vagina), or the fistula is closely related to the distal ureters. The main advantage of a transabdominal approach is that the omentum can be used as a large interposition flap. This makes it the preferred approach for repair of complex, multiple or recurrent fistulae, or when there is a history of pelvic irradiation or coexisting pelvic pathology. It also allows creation of an ileal conduit for urinary diversion in cases that prove to be beyond repair, where patients have been preoperatively counseled regarding such a possible outcome.

The transabdominal approach involves bisection of the bladder from the dome down the posterior wall to the fistula site.[15] Ureteric catheters can be placed to identify and protect the ureters during the procedure. The bladder is mobilized away from the vagina and the fistula excised. The bladder and vagina are then closed separately. The omentum is mobilized and brought down to interpose between them and can be secured into position with one or two sutures if required. Alternative interposition flaps have included pelvic peritoneum,[16] appendices epiploicae or a bladder wall flap.[17]

An extraperitoneal abdominal approach has been reported for the repair of small simple fistulae in which an anterior cystotomy allows access to the bladder and a free bladder mucosal graft is used to close the fistula defect.[18] As exposure is limited with this technique and the use of omentum as an interposition graft precluded, most surgeons favor the intraperitoneal transabdominal approach described previously.

## LAPAROSCOPIC REPAIR

Laparoscopic repair of VVFs was first reported in 1994 by Nezhat *et al*.[19] The technique continues to be developed and aims to achieve success rates equivalent to those for transabdominal open repair whilst avoiding the morbidity of open surgery and shortening postoperative recovery times. The technique involves cystoscopic placement of ureteric catheters and the placement of a catheter through the fistula itself in order to facilitate identification of these structures during laparoscopic dissection. Several studies have shown this technique to be feasible, but the numbers of patients are small and results rely on patient selection and previous laparoscopic experience.[20–22] A review by Kumar *et al*. concluded that, as an alternative to traditional open repair, laparoscopic VVF repair requires good laparoscopic experience, particularly of pelvic surgery and intracorporeal suturing.[23] Laparoscopic VVF repair technique has not gained widespread acceptance due to the technical challenges it poses even to skilled laparoscopic surgeons.

## ROBOTIC REPAIR

The use of robotic technology in the repair of VVF remains extremely limited. There have been reports of roboticassisted laparoscopic repair, in which the advantage of robot is in assisting with intracorporeal suturing during the reconstruction phase of the operation.[24] Initial reports on robotic repair alone suggest successful outcomes and minimal postoperative morbidity, but the number of patients in each series was small.[25,26]

## POSTOPERATIVE MANAGEMENT

Continuous bladder drainage via a urethral catheter is imperative. This ensures that the repair is kept tension-free during healing and allows tissue integrity to re-establish. The placement of an additional suprapubic catheter is advisable in all transabdominal repairs, where the bladder has been opened as part of the procedure. In the postoperative period catheters must be checked regularly to ensure that they remain patent. The advantage of both suprapubic and urethral catheter placement is that bladder drainage is maintained even if one catheter blocks. Inadequate bladder drainage is a common cause of failure of the repair. Bladder spasms can be treated effectively with anticholinergic drugs. There is a concern that these spasms may compromise healing as well as being a source of patient discomfort.[15]

## COMPLICATIONS

The success of VVF repair at the first attempt is approximately 85% for both transabdominal and transvaginal techniques.[11] Complications include recurrent fistula formation, ureteric injury or obstruction, vaginal stenosis, reduced bladder capacity and irritative lower urinary tract symptoms. Small recurrent fistulae can be managed conservatively with prolonged catheterization or a second attempt at surgical repair can be made once the tissues have fully recovered. Vaginal stenosis is rare and may require further surgery to make relaxing incisions or to site skin grafts.

## CONCLUSIONS

VVF remains a condition with devastating physical and social consequences for the patient, regardless of the etiopathology. Their successful management poses a significant challenge. Quick and accurate diagnosis is essential. Timely repair by an experienced fistula surgeon, adhering to fastidious basic surgical principles, will improve outcomes and limit the clinical insult and distress that a VVF invariably causes.
